# The Aging of γδ T Cells

**DOI:** 10.3390/cells9051181

**Published:** 2020-05-09

**Authors:** Weili Xu, Zandrea Wan Xuan Lau, Tamas Fulop, Anis Larbi

**Affiliations:** 1 Biology of Aging Program and Immunomonitoring Platform, Singapore Immunology Network (SIgN), Agency for Science Technology and Research (A*STAR), Immunos Building, Biopolis, Singapore 138648, Singapore; xu_weili@immunol.a-star.edu.sg (W.X.); zandrealau@gmail.com (Z.W.X.L.); 2 Department of Geriatrics, Faculty of Medicine, University of Sherbrooke, Sherbrooke, QC J1K 2R1, Canada; Tamas.Fulop@USherbrooke.ca; 3 Department of Microbiology, National University of Singapore, Singapore 117597, Singapore

**Keywords:** γδ T cells, Senescence, human aging, phenotyping, T cells, markers

## Abstract

In the coming decades, many developed countries in the world are expecting the “greying” of their populations. This phenomenon poses unprecedented challenges to healthcare systems. Aging is one of the most important risk factors for infections and a myriad of diseases such as cancer, cardiovascular and neurodegenerative diseases. A common denominator that is implicated in these diseases is the immune system. The immune system consists of the innate and adaptive arms that complement each other to provide the host with a holistic defense system. While the diverse interactions between multiple arms of the immune system are necessary for its function, this complexity is amplified in the aging immune system as each immune cell type is affected differently—resulting in a conundrum that is especially difficult to target. Furthermore, certain cell types, such as γδ T cells, do not fit categorically into the arms of innate or adaptive immunity. In this review, we will first introduce the human γδ T cell family and its ligands before discussing parallels in mice. By covering the ontogeny and homeostasis of γδ T cells during their lifespan, we will better capture their evolution and responses to age-related stressors. Finally, we will identify knowledge gaps within these topics that can advance our understanding of the relationship between γδ T cells and aging, as well as age-related diseases such as cancer.

## 1. Introduction

Aging research has recently received attention from many parts of the world. This is likely due to the pace of population aging, which is not expected to decelerate within this century [1]. This impending change, which affects both developed and developing countries, will have repercussions on socio-economic and medical systems. The acceleration of aging could be attributed to a few factors: declining birth rates and the baby boomer generation (post world war) have largely unbalanced the population pyramid that encapsulates age distribution; and increased life expectancy due to the advancement of medical technology, vaccination and biomedical research has largely reversed historical mortality rates [2,3,4]. However, the extension of the human lifespan is accompanied by the increased prevalence of chronic diseases such as dementia, heart diseases, sarcopenia and cancer [5,6,7,8,9]. This increased incidence could be due to the accelerating dysfunction of physiological systems, such as the immune system, with age. While vaccines have greatly reduced the mortality of infections such as measles and smallpox [10], hypo-responsiveness and loss of vaccine efficacy observed in the elderly is an obstacle to sustained health, hospitalization and autonomy [11,12,13]. In the context of aging and the immune system, αβ T cells are the most extensively studied as compared to other T cell subsets. This could be due to their abundance in the periphery, which makes the study of αβ T cells more accessible. Their well-characterized roles further imply the physiological importance of age-associated T-cell adaptations, which garners the widespread scientific interest that has contributed to the current depth of T-cell-related investigations [14,15].

T cells can essentially be classified into the adaptive arm, although minority subsets exhibit an innate phenotype. Adaptive T cells include those expressing an αβ T cell receptor (TCR) at their surface and “innate-like” T cells are comprised of T cells expressing a γδ TCR, mucosal associated invariant T (MAIT), invariant natural killer T (iNKT) and germline-encoded mycolyl lipid-reactive (GEMT) [16]. This classification is based on their response speed when encountering new antigens and the ability to form memory cells that persist in long-term immunosurveillance. In this review, we will focus on γδ T cells and their contextual importance in cancer immunosurveillance, and on the reactivation of latent infections such as tuberculosis and virus-infected cells, as these topics require consolidation in the literature but are often neglected in the aging context.

First, we will describe γδ T cells and their respective ligands in both mice and human. Next, we will analyze γδ T cells from cradle to grave (i.e., development to old age) to understand their aging trajectory during lifespan. Finally, we will suggest future developments that are necessary for our comprehension of how γδ T cells subsets are affected during the aging process. A better understanding of γδ T cell biology should enable scientists to tailor optimized immunotherapy that targets age-associated immune impairments in the future.

## 2. Immune System

The immune system is the host natural defense system against foreign pathogens such as bacteria, fungi and viruses. The features of the innate immune systems include a fast response time, non-specific reaction to a particular antigen and no long-term memory properties [17]. However, in recent years, the paradigm that the innate immune system lacks a memory feature has been debated due to the emerging concept of trained immunity observed in myeloid cells [18]. Immune cells that are categorized under the innate immune system include neutrophils, monocytes, macrophages, natural killer cells (NK cells) and innate lymphoid cells (ILCs). As for the features of the adaptive immune system, it includes a slow response time upon encountering a new antigen, specificity to a particular antigen and long-term memory properties that enable it to respond in a much faster fashion when the organism next encounters the same antigen. Immune cells that are categorized under the adaptive immune system are namely T cells and B cells. However, recent studies have shown that within the T and B cells, there are subpopulations of T and B cells that have features similar to the innate immune system. These subpopulations are the γδ T cells, MAIT cells, iNKT cells and innate-like B cells [19]. Thus, with the concept of trained immunity observed in myeloid cells and unconventional T and B cells having certain features of innate immune cells, the categorization of certain immune cells into innate and adaptive immunity is no longer as well defined as before and will be dependent on the exact features we utilize to classify them.

While it is important to have both innate and adaptive components in the immune system to provide a holistic defense system against foreign pathogens, communication between immune cells is essential. Molecules that facilitate such communication include cytokines, chemokines and surface receptors that enable immunes cells to transmit signaling and also be responsive to the current environment [20]. Therefore, we will also discuss these aspects and how this may provide evidence for supporting eroded immunity in aging. 

## 3. Immunity in Aging Humans

Numerous clinical studies have shown that aging is associated with increased susceptibility to viral, bacterial infections, cancer, and reduced vaccine efficacy [14]. In addition, several age-related diseases arise, such as dementia and heart disease in the elderly [21]. These increased incidences of diseases observed in elderly individuals could be attributed to the immune system being dysfunctional with age and this is often termed as immunosenescence [22]. Major aspects of immunosenescence include inflamm-aging (chronic low-grade sub-clinical inflammation), reduced ability to fend off infections and reduced response to new antigens [23]. Whether these age-related observations are due to aging per se or the continuous stimulation of the immune system is still a debate. At the molecular level, it is likely that the immune cells are similarly susceptible to genomic instability, increased inflammation and increased oxidative stress affecting major cellular functions including proteostasis, mitochondrial function and damage repair, which then could result in reduced immunosurveillance and healthspan of the host [24].

The innate immune system in older adults is associated with two main features: (i) increased inflammation in the immune system (ii) immune paralysis when specific functions are required [25]. It has been shown that cells involved with age-associated erosion have up-regulation of pro-inflammatory cytokines and associated receptors, with a concomitant decrease in effector functions such as phagocytosis and free radical production during steady-state [26,27]. Besides that, one of the default roles of innate immune cells is to prime the adaptive immunity to eliminate the aggressors. For instance, antigen presentation is impacted during aging as it seem that dendritic cell activation of the CD4+ T cells is less efficient in the elderly [28]. This may be likely due to altered TCR-dependent signaling caused by old age [29]. Besides antigen presentation by the dendritic cells, the increased production of pro-inflammatory cytokine by innate immune cells such as monocytes and macrophages may also influence CD4+ T cell reactivity [30]. In the aging adaptive immune system, many alterations have been reported. The main characteristics of B cell immunosenescence include decreased B cell lymphocytes with a shrinkage in B cell repertoire, the decreased quality of antibody response during an immune response, and an increase in autoantibodies, as well as a reduced response to vaccination in the elderly, likely due to intrinsic B cell defects [31,32,33]. The characteristics of T cell immunosenescence include shrinkage of the TCR repertoire due to the decrease in naïve T cells (following natural thymic involution) and a concomitant increase in memory T cells, some of which exhibit an exhausted and/or senescent profile [34]. αβ T cells form the majority of the T cell repertoire and can be classified into CD4 helper and CD8 cytotoxic T cells. However, besides the αβ T cells, the γδ T cells, mucosal associated invariant T cells (MAIT) and invariant natural killer T (iNKT) cells also exist. These non-classical T cells are lower in frequency in the circulation but occur in higher frequencies in the tissues [35,36,37]. There are many factors that could explain the decrease in immunity in older adults and one of them is T cell exhaustion. This phenomenon emerges after T cells have undergone repeated or chronic stimulations over the course of the host lifespan. The continuous stimulation results in the progressive loss of effector functions, reduced cytokines production due to the emergence of inhibitory receptors such as PD-1, CTLA4 and LAG3 [38]. This is observed in the context of cancer immunotherapy, in which targeting these receptors enables recovery of the above-mentioned effector functions. The other factor involved is natural thymic involution, which is the shrinkage of the thymus, defined as an irreversible decline in the size and function of the thymus, that accounts for some of the major characteristics of T cells’ immunosenescence. In old age, thymic involution is subjected to hormonal control and the generation of naïve T cells continues at a reduced rate, with altered patterns and decreased emigration to the periphery [39,40]. Due to the deficiency of generating new and functional naïve T cells, this could then result in an increased risk of severe infection and incidence of cancer in the elderly due to an overall reduction in T cell immunosurveillance in elderly individuals. While thymic involution may have been evolutionarily programmed to reduce the wastage of biological reserves (as a majority of naïve T cells are eliminated during maturation), especially by mid-life, as the majority of pathogens may have already been encountered for the host to survive past reproduction age, this process could have limitations in populations with an extended lifespan due to advancements in medical technologies.

## 4. Gamma Delta T Cells Subsets

Since the evolution of jawed vertebrates (over ~450 million years of evolution), γδ T cells have existed as one of three main lymphocyte lineages that perform immune surveillance alongside αβ T cells and B cells [41,42]. 

In mice, γδ T cell subsets have been highlighted as bearing semi-invariant TCRs, and recognizing a limited range of self-ligands [43,44,45]. These subsets are typically grouped according to the Vγ chain they express, which is, in turn, associated with their tissue of residence [46]. The Vγ1+ (Helig and Tonegawa’s System) subset represents one of the major subsets in the circulation of mice and is associated with the production of various cytokines such as IL-4 and IL-17 [47,48]. In the mouse epidermis, dendritic epidermal T cells (DETC) mostly consist of the Vγ5+Vδ1+ subset, which is the most extensively studied γδ T cell subset in mice. The Vγ5+Vδ1+ subset displays an “innate-like” property and is able to respond to specific stress-associated TCR ligands, produce cytokines such as IFN-γ and play a role in wound healing [49,50,51]. The Vγ6+ subset produces IL-17 and IL-22 [52], and is most often paired with Vδ1. The Vγ6+Vδ1+ subset, which could migrate to the genital tract and lungs, seems to respond to inflammation and may play an immune-regulatory role during pregnancy [53,54]. In addition, it has also been shown to be able to inhibit the development of pulmonary fibrosis by exerting antibacterial activities in the lung [55]. 

In humans, γδ T cells constitute a minor subset among T lymphocytes, constituting 1%–10% of mature circulating T cells [56]. Unlike the majority of αβ T cells, most γδ T cells (>70%) are CD4-CD8-, some (~30%) are CD8+CD4- and very few (<1%) are CD4+CD8- [57]. These cells can be classified as “innate”, although they have recently been discovered to possess “adaptive” features. [58,59,60] This duality of γδ T cell biology was attributed to a combination of their non-MHC-restricted antigen specificity and capacity to mount rapid immune responses to a wide range of tissue stressors. [61,62]. Similar to αβ T cells and B cells, the structural diversity of γδ T cells is dependent on V(D)J somatic recombination, which generates a set of highly diverse receptors for antigen recognition. However, the repertoire of γδ T cells is limited as compared to αβ T cells and B cells. This diversity is mainly generated in the complementary-determining region 3 (CDR3) of the TCR [63], where a variety of γδ T-cells are generated from a combinatorial union of the δ and γ chain.

**Figure 1:**
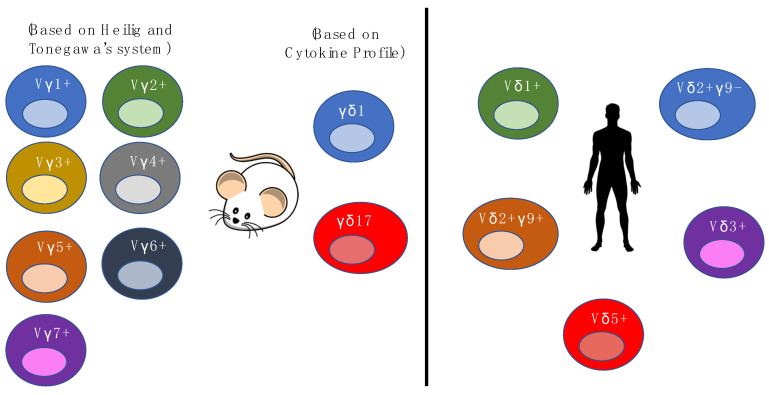
Illustrations of the different γδ T cell populations in mice and human and how γδ T cells are categorized.

Human γδ T cells are generally divided into four populations based on their TCR δ chain expression, namely the δ1, δ2, δ3, and δ5 populations [64,65]. The Vδ gene segments in humans that are most frequently used in the rearrangement of the δ chain are Vδ1, Vδ2 and Vδ3 [66,67]. Vδ3+ cells, which are found in the liver and gut epithelium, often pair with Vγ2 and Vγ3, and are also known to expand during cytomegalovirus (CMV) activation and B cell chronic lymphocytic leukemia [68,69,70]. The majority of Vδ1+ cells reside in the gut, liver and other epithelial tissues, and pair with Vγ2, Vγ3, Vγ5, Vγ8, and Vγ9 chains [71,72]. Recent studies have shown that the Vδ1+ that are highly enriched in the gut, preferentially pair with Vγ4, express Nkp46+, have a cytotoxic phenotype and are depleted upon gluten-induced inflammation [73,74]. Vδ1+ cells have been shown to be MHC-independent and involves a highly adaptive yet unconventional form of immunosurveillance. Vδ1+ cells are implicated in immune responses to viral infections such as CMV, and are able to recognize cancerous cells, as well as react to stress-induced molecules, such as MHC class I-related chains A and B (MICA and MICB) that are expressed on viral infected cells [49,52,75,76]. On the other hand, the Vδ2 chain often combines with the Vγ9 chain to form the Vγ9+Vδ2+ subset and this subset is the most abundant γδ T cells in the peripheral blood of adults [77]. The Vγ9+Vδ2+ subset is able to aid in both anti-microbial immune responses and αβ T cell responses. As for Vγ9-Vδ2+, recent studies have shown that this population is similar to Vδ1+, as it adopts an “adaptive” biology—it can clonally expand, differentiate and respond to CMV infection. Vγ9+Vδ2+ T cells are more “innate-like” in comparison, due to the contrast in undergoing clonal amplification and differentiation relative to Vδ1+ subsets. As most studies have focused on Vδ1+ and Vδ2+ subsets, information on Vδ3+ and Vδ5+ subsets are lacking, even though they are present in the peripheral blood. The functional diversity of γδ cells can be better appreciated if further research can shed light on whether these subsets are more “innate-like” or “adaptive-like”. Overall, current findings show that γδ T cells have different physiologic roles depending on their molecular nature and their location. A deeper understanding of γδ T cell biology is necessary to fully delineate their roles in organ and tissue structures (Figure 1).

## 5. Ligands

### 5.1. In Mice

γδ T cells are able to recognize a wide range of molecules and most γδ T cells ligands are non-polymorphic in nature. Unlike αβ T cells, γδ T cells do not require the help of MHC class I and class II molecules for the recognition of antigens. In mice, their TCR and activating receptor, NKG2D, are able to recognize ligands such as H60, MULT-1, RAE-1 [78] Qa-1 and MHC-like T10 and T22 [79,80,81]. Crowley et al. stated that both T10 and T22 molecules are shown to be activation-induced and confers specificity to ~0.4% of the γδ T cells in normal mice [82]. Moreover, the MHC-like T22 ligand was found to bind to the γδ TCR G8, where G8 uses germline-encoded residues of the δ chain of CDR3 loop to bind to T22. T10, conversely, has a weak affinity towards G8 [83]. Skint-1, a butyrophilin-like molecule, is also a ligand that mice Vγ5+Vδ1+ DETCs recognize. Skint-1, expressed by thymic epithelial cells and keratinocytes, is important for the maturation of mouse thymocytes and the appearance of Vγ5+Vδ1+ DETCs in the epidermis [84]. Evidence have shown that Skint-1 specifically drives the development of the DETC compartment, but the molecular mechanisms behind Skint-1 activity are unclear [85]. Furthermore, cardiolipin, a ligand that can bind to CD1d, is recognized by mice γδ T cells, and CD1d has is expressed on both mice and human γδ T cells [86].

### 5.2. In Humans

In humans, γδ T cells—more specifically Vδ1+ and Vδ3+ γδ T cells—have demonstrated the ability to recognize lipid-based antigens presented by CD1d [87,88]. CD1d binds to the Vδ1 TCR mainly through the CDR1δ loop, and antigen specificity is dictated by the CDR3γ loop [89]. Besides CD1d, Vδ1+ cells can also be activated by glycolipids presented by CD1c on the surface of immature dendritic cells, and this interaction induces dendritic cell maturation and the production of IL-12 [90,91]. Additionally, the Vδ1+ subset also recognizes MHC-related molecules: MICA and MICB, although MIC, which is upregulated in infected cells, is uninvolved in antigen presentation [92,93,94,95]. With regards to the binding mechanism, the recent elucidation of the structure of a MIC-reactive Vδ1 TCR suggests sequential recognition of MIC by TCR and NKG2D [96]. Thus, the NKG2D receptor is crucial for Vδ1+ cytotoxicity against various carcinomas [97]. The Vδ2+ subset, on the other hand, recognizes different ligands. By far, the most potent compound that the Vγ9+Vδ2+ subset responds to is the microbial metabolite (E)-4-hydroxy-3-methyl-but-2-enyl pyrophosphate (HMB-PP), which is produced by many bacterial strains, malarial parasites and *Toxoplasma gondii* [98]. The Vγ9+Vδ2+ subset is also able to react to other phosphoantigens, such as isopentenyl pyrophosphate (IPP) and dimethylallyl pyrophosphate (DMAPP), which are derived from both the mevalonate [99] and 2-C-methyl-D-erythritol 4-phosphate (MEP) pathways of isoprenoid metabolism in many bacteria and parasites [100]. IPP plays an essential role in mediating immunity against pathogens and also has potent anti-tumor activities, as tumor cells that produce elevated concentrations of IPP are recognized and killed by Vγ9+Vδ2+ cells [101,102]. The latter relies on features such as MHC unrestricted killing of tumor cells, antibody-dependent cellular cytotoxicity, and effector mechanisms that rely on cytokine release [103]. 

## 6. Gamma Delta T Cell Subsets During Lifespan

### 6.1. In Mice

**Figure 2:**
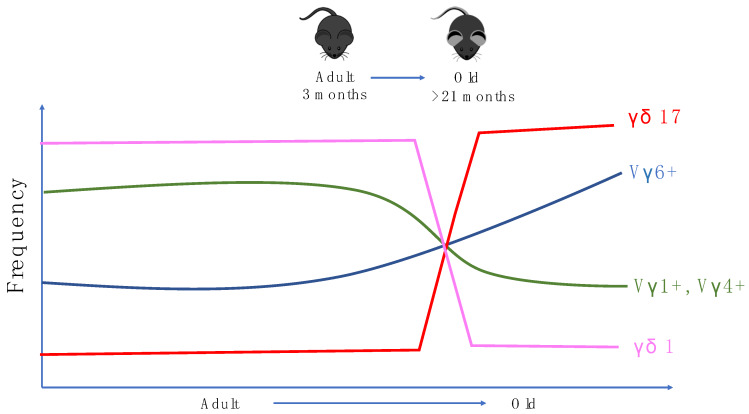
Alterations in the cytokine profile and γ chain utilization of mice γδ T cells in peripheral lymph nodes with age.

In mice, γδ T cells are the first T cells to leave the thymus. Vγ5+Vδ1+ DETCs are the first T cells to be developed before birth and bear invariant TCRs [104]. This is followed by the production of IL-17 producing Vγ6+Vδ1+ T cells which can be found in many tissues such as the lung, liver and intestinal lamina propria [105,106,107]. After birth, more diverse γδ T cell populations using Vγ4, Vγ1, and Vγ7 chains are produced and found in the circulation and other parts of the tissues. Mouse γδ subsets have been suggested to have an innate-like biology. However, there is evidence in multiple models which suggests that IL-17 producing Vγ6+ T cells and Vγ4+ T cells (γδ17 T cells) undergo adaptive-like differentiation through naïve precursors into mature γδ17 T cells in peripheral lymphoid organs [108]. In terms of aging, Chen et al. demonstrated that aging alters TCRδ chain usage and the clonal structure of γδ T cells. This study demonstrated that in aged mice, the utilisation of Vδ6 in Vγ1+ γδ1 T cells increases slightly while Vδ2 is less favored. In Vγ4+ γδ1 T cells, usage of Vδ7 was also slightly reduced, together corroborating the observation that δ chain utilization is altered by aging in ice. More importantly, this study shows that in aged mice, γδ17 T cells constitute the majority of the γδ T cell pool in the lymph nodes of aged mice as the γδ17 T cells population increases from 15% to around 60%–80% among total γδ T cells. Moreover, γδ1 T cells and their precursors have reduced frequencies during aging [109]. Interestingly, in humans, there is also a shift in Vγ/Vδ usage during aging [110], indicating some parallels in age-related γδ biology in both mice and humans (Figure 2).

### 6.2. In Humans

**Figure 3:**
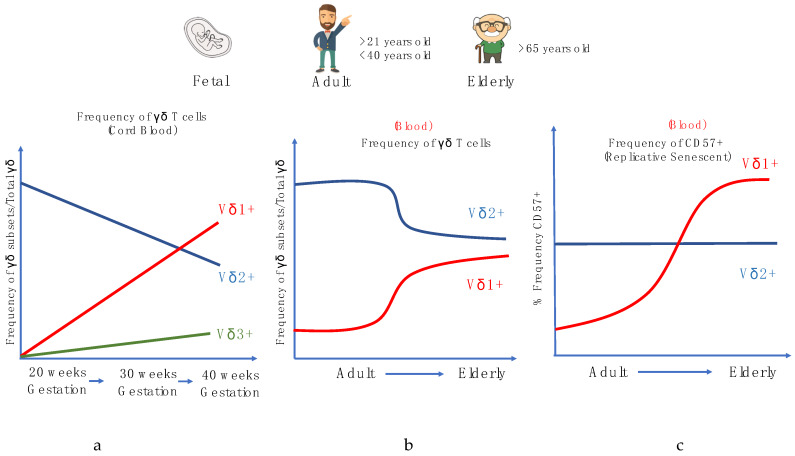
Alterations in human γδ T cells during development and aging. (**a**) Frequency of γδ subsets/Total γδ in the cord blood during gestational weeks, (**b**) Frequency of γδ subsets/total γδ in the periphery with age. (**c**) Frequency of CD57+ γδ cell populations in the periphery with age.

In humans, during the gestational phases, the development of γδ T cells primarily occurs in the fetal thymus, and different subsets arise through rearrangements at distinct phases of thymic development. γδ TCR gene rearrangement can be detected by embryonic day 14 in the mouse thymus, week 8 in humans, and canonical subsets can also be detected extrathymically in both species during fetal development [111,112,113]. In the human fetus, the Vγ9+Vδ2+ subset is among the first T cell subset to be developed and this population further expands during childhood, although these cells have a distinct lineage, as recent studies have shown that the ontogeny between fetal blood γδ and adult blood γδ is dissimilar [112,114,115,116]. Vγ9 and Vδ2 V gene segments can be detected as early as 5 to 6 weeks gestation in the fetal liver and after 8 weeks in the fetal thymus [117]. By mid-gestation (20 to 30 weeks), the Vγ9+Vδ2+ subset dominates the γδ repertoire and is capable of producing IFN-γ in response to HMB-PP stimulation. At birth (~gestational week 40), the Vγ9+Vδ2+ constitutes a smaller proportion as the Vδ1 repertoire increases, with the Vγ9-Vδ1+ comprising the majority of the γδ repertoire, and Vδ3 making up the remaining proportion of γδ T cells in cord blood [77] (Figure 3a). 

Often in human aging studies, various phenotypic markers, such as CD27, CD45RA, and CD57, are widely used to study the differentiation of αβ T cells. There are four stages of differentiation: the ‘Naïve’ CD45RA+CD27+, Central memory CD45RA-CD27+, Effector memory CD45RA-CD27- and, lastly, the Terminal effector CD45RA+CD27- T cells, with the terminal effector often expressing the senescence marker CD57. Using these surface markers, αβ T cells are known to have a higher frequency of highly differentiated and replicative senescent T cells in circulation with aging, denoted by the surface marker CD57 that implies impairment in their ability to proliferate [118]. However, our recent studies, along with others, have shown that while pan-γδ T cells do not express the same phenotypic changes as αβ T cells during aging [119,120]; the frequencies of γδ T cell subsets, particularly the Vδ2+ subset, decrease with age after age 30. However, the extent of the latter is influenced by sex and nationality [121,122,123,124] (Figure 3b). Furthermore, it was suggested that Vδ2+ T cells are more resilient to cellular aging and environmental stress as compared to αβ T cells and other γδ T cells subsets. Conversely, Vδ2- (Vδ1+ and Vδ1-Vδ2- cells) were found to respond similarly to CD8+ αβ T cells, in that we observed a decrease in the frequencies of naïve populations and higher frequencies of memory/effector phenotypes, in response to CMV and lifelong immune stress. It was also shown that, during aging, the CD57 senescence marker is more commonly expressed in Vδ2- γδ T cells but not Vδ2+ cells (Figure 3c). This observed trend in the Vδ2- γδ T cells is similar to many aging studies of αβ T cells, and proliferation assays have been conducted to show that CD57 is a universal marker of replicative senescence for both αβ T and γδ T cells. 

The differential impact of aging on Vδ1+ and Vδ2+ could be due to their distinct response after stimulation. It has been shown that while IL-15 stimulation is able to proliferate Vδ1+ CD27-low [58], it induces a high cell death rate on Vδ2+ TEMRA with low anti-apoptotic Bcl-2 expression [125]. This suggests that highly differentiated Vδ2+ might not be able to accumulate with lifelong stressors due to this unique homeostatic mechanism, granting them the resilience to maintain its function during aging. In twin studies, investigations on the subset specificities of Vδ1+ and Vδ2+ demonstrate higher heritability within the Vδ2+Vγ9+ population, while Vδ1+ T cell diversity is more dependent on the environment [126]. Together, these data could suggest that the phenotype of Vδ2+ in an individual remains relatively stable during aging [127].

However, while the frequencies of CD57+ Vδ2+ do not generally increase with aging, a recent study by Bruni et al. 2019 shows that elderly (>60 years old) liver metastatic colorectal cancer patients reveal significantly higher frequencies of CD57+ Vδ2+, in both the periphery and the liver, after undergoing chemotherapy, as compared to younger patients [128]. This could imply that the Vδ2+ in the elderly could still be vulnerable to external stressors such as chemotherapy, infections and radiotherapy. However, the mechanisms that contribute to this susceptibility remain unknown and should be pursued in the future to benefit custom immunotherapies for age-related disease. 

## 7. Gaps in Aging Research Related to γδ T Cells

### 7.1. Homeostasis of γδ T Cells in Tissues

While most studies reported data on peripheral γδ T cells in humans, γδ T cells are found in higher frequencies in tissues and organs as compared to the periphery. Thus, it is also important to investigate how the functional capacity of γδ T cells modulate with age in these compartments. In mice, some studies highlighted age-related changes in γδ T cells in the lymph nodes and other tissues. However, within the various organs, there is little effort to comprehensively profile phenotypic changes in γδ T cells during aging. This knowledge gap must be addressed for us to fully understand how aging impacts the γδ T cells as a whole in the murine immune system, which is more adaptable to study than the human system. In humans, there are even fewer studies in tissues and aging—which could be related to their poor accessibility to donors. A recent study by Hunter et al 2018 reveals that the phenotype of Vδ2- γδ in the liver is largely CD27-/low despite being CD27hi in the periphery of the same individuals [129]. As demonstrated for classical αβ T cells, this finding illustrates that the maturation and differentiation phenotype of cell populations shows poor congruency within different organs and tissues [130]. While these studies are challenging to conduct as biopsies are rare, it is nonetheless important to investigate the skin, gut and lung, as these are frontiers where the immune system first encounters foreign pathogens [131]. Given that γδ T cells have the tremendous immunosurveillance potential to defend against virus-infected and cancerous cells, an understanding of the functionality of γδ T cells in organs and tissues and how γδ T cells change with age in terms of frequency, cytokine secretion, cytotoxic capacity, proliferation, chemotaxis and location will be crucial to improve γδ T cell immunotherapy in elderly individuals. 

### 7.2. The Inhibitory Receptors on γδ T Cells and Cancer Immunotherapy

In recent years, immunotherapy has revolutionized the way we treat cancer and been recognized as one of the four pillars of cancer treatment alongside surgery, chemotherapy and radiotherapy [132,133]. Current immunotherapy mainly relies on antibodies that block the ligation of inhibitory receptors such as PD-1, allowing tumor-infiltrating CD8 T cells to exhibit its cytotoxicity capacity against tumor cells. This approach is effective to a certain extent, as one of the immunoevasive strategies that tumor cells utilize is to express PD-L1 on their surface to ligate with PD-1, rendering CD8 T cells ineffective [134]. While anti-PD-1 immunotherapy has gained the spotlight due to its prominence in the literature, the neutralization of other inhibitory receptors such as LAG3 and CTLA4 has been proposed for monotherapy and combinatorial therapy in different clinical trial settings to fine-tune cancer immunotherapy. These novel approaches are necessary as anti-PD-1 therapy has limited efficacy—while revolutionary in terms of promising an increase in five-year survival rates, it has not been effective in some patient and cancer settings [135,136,137]. However, checkpoint inhibitor studies are mostly focused on CD8 T cells and have neglected γδ T cells, despite their capacity for cancer immunosurveillance. The risk of cancer is also exacerbated by aging and γδ T cells possess a cytotoxicity capacity for the elimination of tumor cells. Furthermore, the infiltration of γδ T cells in tumors is prognostic to a certain extent in cancer patients [138]. Therefore, the age-associated expression of various inhibitory receptors, such as PD-1, LAG-3 and CTLA-4 on γδ T cells should be studied in peripheral blood or tumors for more immunotherapy specificity.

### 7.3. Cytokines, Chemokines and γδ T Cells

Cytokines and chemokines are soluble factors that are secreted by cells to communicate among themselves, contributing to downstream effects such as activation, proliferation and recruitment, which are essential to resolving an infection. Studies have shown that γδ T cell subsets are able to respond to IL-12, IL-15, IL-18 which then leads to activation, the secretion of cytokines and proliferation [139]. In aging studies on αβ T cells, the authors have shown that elderly T cells display attenuated tyrosine phosphorylation of the protein tyrosine kinase ZAP-70, LAT and PLCγ due to TNFα and reduced phosphorylation of STAT3 and STAT5; this is possibly due to the increased expression of suppressor of cytokine signaling 3 (SOCS3) caused by IL-6. The latter suggests that the inflamm-aging environment often observed in elderly individuals has an impact on the signaling pathways of αβ T cells [140]. However, whether the γδ response to various cytokines during inflammaging, such as IL-12, IL-15 and IL-18, is altered with aging, requires investigation. 

Chemokine receptors are essential for the migration and recruitment of cells to the site of demand. In aging studies conducted on αβ T cells, there is a change in Th1 (cells that express CXCR3) to Th2 (cells that express CCR4 and CRTH2) ratios [141] and altered ratios of Th17 (cells that express CCR6) to Treg cells in the periphery [142]. This phenomenon may be related to changes in the chemokine receptor expression of T cells with age. Studies have shown that γδ T cells subsets are able to express CXCR3, CCR5, CCR6, CCR7, CX3CR1, which react to IP-10, CCL3/4, CCL20, CCL21 and CX3CL1, respectively. However, whether their expression and migratory capacity is stable during aging has not been investigated. Having this knowledge will then allow us to fully appreciate changes in the functionality of γδ T cells subsets during aging.

### 7.4. γδ T Cells, Respiratory Diseases and the Utility of Vaccination

Age is a major risk factor for mortality resulting from respiratory diseases such as influenza, pneumonia, chronic obstructive pumolnary disease (COPD), the recent coronavirus-induced disease 19 (COVID19) and cardiovascular diseases [143,144,145,146,147]. While some studies describe the protective roles of γδ T cells in influenza infections [148,149,150,151], klebsiella pneumonia [152], cardiac γδ T cells in dystrophin-deficient mice [153], and also the distribution of γδ T cells in COPD [154], this type of study does not involve the concept of aging. It is therefore essential to study the execution of these roles in the aging context to understand whether γδ T cells remain functional and protective in old age.

As immune-related pathologies are the main cause of death in the very old, especially pulmonary infections, a better understanding of the roles of lung-resident γδ T cells may be crucial. As observed in the recent severe acute respiratory syndrome coronavirus 2 (SARS-CoV-2) pandemic, the majority of individuals who experience complications and severe COVID-19 symptoms are the elderly. The age-associated deficiency in respiratory function has been a major contributing factor to this life-threatening scenario, and is associated with an inflammatory milieu and accompanying tissue damage. γδ T cells have been shown to be the first cells to migrate to the lung in response to tuberculosis (TB) infection and support the life-long surveillance of TB-associated granuloma [155]. Pneumococcal infections, which can contribute largely to death in old age, can be controlled by the pool of non-conventional cells, including γδ T cells [156]. The upper respiratory tract is colonized by Streptococcus pneumoniae but this remains asymptomatic in healthy individuals. It is likely the combination of the host (lifestyle habits, immune status) and pathogen status (virulence) that determines if this silent colonization may evolve to manifest mild or moderate symptoms such as sinusitis and pneumonia. Lung-resident γδ T cells are involved in respiratory infections. For instance, their activation by endogenous mevalonate metabolites or via IL-17 is crucial for the clearance of pathogens. 

Whether impaired immunity in old age could explain the higher susceptibility to COVID-19 symptoms is an important avenue to test, and γδ T cells may be primordial in this context. Vaccines have helped to prevent epidemics and pandemics in the past century. However, in elderly individuals, reduced vaccine efficacy is a major hurdle to achieving longer healthspan. The emerging role of γδ T cells in vaccine-mediated protection from infectious diseases has been elegantly reviewed in [157] but many studies have not investigated the role and functionality of γδ T cells during the vaccination of elderly individuals to assess if these functions are preserved with age. A recent study by Stervbo et al. has shown that there are kinetic age-dependent differences after influenza vaccination in γδ T cells. The authors showed that during the time course of influenza vaccination, disturbances in the absolute counts and frequencies of CD38+ γδ were more dynamic in the young than the old [158]. However, as γδ T cell subsets are impacted differentially during aging, it will also be important for future studies to separate γδ T cells into Vδ1+ and Vδ2+ for greater clarity and resolution. Nonetheless, a general γδ survey could offer a first glimpse of the impact of aging on γδ T cells during vaccination.

## 8. Conclusions

Biomedical research on human aging has assumed unprecedented importance with the looming silver tsunami, and its impact could be seen in the next 10 to 30 years. Healthcare remains a challenge for the elderly and understanding the dysfunctionality of the immune system with age could be key to improving health outcomes. However, in order to harness the potential of the immune system, and in particular the γδ T cells, more research needs to be done in order to better understand how γδ T cells subsets change or are dysregulated with age in the different tissue compartment. It is also important to functionally assess human γδ T cells with respect to cytokine secretion, cytotoxic capacity, proliferation and chemotaxis in response to various stimuli and ligation of inhibitory receptors to understand how they change in an in vivo natural infection, as parallels to animal models are lacking. While aging studies on human γδ T cells are rare, human γδ T cells are an attractive candidate that can be targeted to eliminate tumor- and virus-infected cells, which are more prevalent in the elderly. Understanding the functional and phenotypical modulations of γδ T cells with age is the first step necessary for their reversal, providing a window of opportunity to improve medical outlook within the elderly. 

## Abbreviations

CCL	C-C Ligand
CCR	C-C Chemokine Receptor
CD	Cluster of Differentiation
CDR	Complementary Determining Region
CMV	Cytomegalovirus
CTLA-4	Cytotoxic T Lymphocyte Activation 4
COPD	Chronic Obstructive Pulmonary Disease
COVID19	Coronavirus Induced Disease 19
CXCR	CXC Chemokine receptor
CX3CR1	CX3C chemokine receptor 1
CX3CL1	CX3C Ligand 1
DETC	Dendritic Epidermal T Cells
DMAPP	dimethylallyl pyrophosphate
GEM	germline-encoded mycolyl lipid-reactive T
HMB-PP	(E)-4-hydroxy-3-methyl-but-2-enyl pyrophosphate
IP-10	Interferon gamma-induced protein 10
IPP	isopentenyl pyrophosphate
MHC	Major Histocompatibility Complex
MIC	MHC Class I-related
IFN	Interferon
IL	Interleukin
ILC	Innate Lymphoid Cell
LAG3	Lymphocyte-activation gene 3
iNKT	invariant Natural Killer T
MAIT	Mucosal Associated Invariant T
NK	Natural Killer
PD1	Programmed Cell Death Protein 1
PD-L1	Programmed Cell Death Ligand 1
SARS CoV-2	Severe Acute Respiratory Syndrome Coronavirus 2
SOCS	Suppressor of cytokine signaling
TB	Tuberculosis
TCR	T Cell Receptor
Th	T Helper
TNF	Tumor Necrosis Factor
